# Increased serum level of Lp-PLA2 is independently associated with the severity of coronary artery diseases: a cross-sectional study of Chinese population

**DOI:** 10.1186/s12872-015-0001-9

**Published:** 2015-02-26

**Authors:** Anping Cai, Guang Li, Jiyan Chen, Xida Li, Liwen Li, Yingling Zhou

**Affiliations:** Department of Cardiology, Guangdong Cardiovascular Institute, Guangdong General Hospital, Guangdong Academy of Medical Sciences, 106Zhongshan Road 2, Guangzhou, 510080 China

**Keywords:** Coronary artery disease, Lipoprotein-associated phospholipase A2, Atherosclerosis

## Abstract

**Background:**

Lipoprotein-associated phospholipase A2 (Lp-PLA2) plays complex and adverse roles on atherosclerosis. Current study was to investigate whether increased plasma Lp-PLA2 level is independently associated with the severity of coronary artery diseases (CAD).

**Methods:**

Totally 781 participants were enrolled and performed coronary angiography (CAG) to figure out the number of coronary artery stenosis. According to clinical presentation, electrocardiography, cardiac biomarker, and CAG result, participants were divided into control (excluded CAD), stable angina (SA), unstable angina (UA) and acute myocardial infarction (AMI) groups. Baseline characteristics were recorded. Statistical analyses were performed to evaluate the relationship between Lp-PLA2 level and CAD severity.

**Results:**

Plasma levels of Lp-PLA2 in control, SA, UA and AMI groups were 7.38(3.33-9.26) μg/L, 5.94(2.89-8.55) μg/L, 8.56(5.34-11.95) μg/L and 8.68(5.56-13.27) μg/L respectively (P < 0.001). After adjusted for age, gender, smoking, diabetes mellitus, hypertension, low-density lipoprotein-cholesterol (LDL-C), high-density lipoprotein-cholesterol (HDL-C), apoprotein A (apoA) and statins, Lp-PLA2 level was still independently associated with CAD severity, with odd ratio (OR) of 1.055 (AMI group versus control group, 95% confidence interval (CI) 1.021-1.090, P < 0.05). Additionally, the relationship between Lp-PLA2 level and the number of stenosis coronary artery was also assessed. Lp-PLA2 levels in control, single-vessel, and multiple-vessels stenosis groups were 7.38(3.33-9.26) μg/L, 7.80 (4.05-10.76) μg/L and 8.29(5.18-11.76) μg/L respectively (P for trend < 0.001). After adjusted for age, gender, smoking, diabetes mellitus, hypertension, LDL-C and HDL-C, apoA and statins, Lp-PLA2 level remained independently associated with the number of coronary artery stenosis, with OR of 1.053 (multiple-vessels stenosis group versus control group, 95% CI 1.025-1.069, P < 0.05).

**Conclusion:**

Increased Lp-PLA2 level is independently associated with CAD severity, and Lp-PLA2 level may be used to discriminate those who are at increased risk of cardiovascular events.

## Background

Atherosclerotic cardiovascular disease (ASCVD) is still the leading cause of morbidity and mortality worldwide despite great advances on diagnosis and treatment have been achieved in the past decades [1]. Knowingly, inflammation plays critical and continuous roles on the initiation and progression of atherosclerosis and ASCVD [2,3], and increased serum level of inflammatory biomarker such as high sensitivity C-reactive protein (Hs-CRP) has been recognized as an important predictor for cardiovascular diseases risk [4,5].

Lipoprotein-associated phospholipase A2 (Lp-PLA2) is an enzyme excreting predominantly from atherosclerotic plaques by macrophages and neutrophils and then circulating in blood stream [6]. Previously, clinical epidemiological studies showed that increased plasma level of Lp-PLA2 was associated with increased risk of cardiovascular events such as myocardial infarction and ischemic stroke [7-9], and Lp-PLA2 inhibitors could significantly reduce the incident of cardiovascular events as compared to placebo treatment [10,11]. Data from basic experiments further supported clinical findings [12-14]. For example, in animal model of hyperlipidemia and hyperglycemia, as compared to placebo treatment, Lp-PLA2 inhibitor reduced macrophage accumulation, diminished necrotic lipid-core volume and thickened fibrous cap of coronary atherosclerotic plaque [14]. Therefore, Lp-PLA2 has been incorporated into cardiovascular diseases risk assessment algorithm [15]. Nevertheless, despite striking and encouraging findings from previous studies, two recent large randomized, controlled and prospective clinical trials showed that darapladib treatment, a selective Lp-PLA2 antagonist, resulted in no better improvement of clinical outcomes in patients with stable or unstable coronary artery diseases (CAD) [16,17].

In light of the conflicting findings of previous studies, we conducted a cross-sectional and observational clinical study to investigate whether increased plasma level of Lp-PLA2 is independently associated with the severity of CAD, and we believe that the clinical implication of our study would add, if any, valuable information to address whether Lp-PLA2 could be used to predict CAD risk in the future.

## Methods

### Studied subjects and protocols

Studied subjects were enrolled from January of 2013 to April of 2014 after informed consent was obtained, and our current study was approved by the Ethical Committee of Guangdong General Hospital. Coronary angiography was performed to figure out the number of coronary artery stenosis in terms of single or multiple vessels stenosis (≥50% stenosis). Those without significant coronary artery stenosis (<50% stenosis) were defined as control group. Clinical presentations in terms of stable angina (SA), unstable angina (UA) or acute myocardial infarction (AMI) were diagnosed according to clinical manifestation, electrocardiography findings and cardiac biomarkers by two experts of cardiology. Demographic characteristics including age, gender, family history of ASCVD, smoking status, hypertension and diabetes mellitus were recorded by questionnaire and were double-checked by two experts of cardiology. Fasting venous blood at admission was drawn for the assessment of fasting blood glucose (FBG), total cholesterol (TC), triglyceride (TG), low density lipoprotein-cholesterol (LDL-C), high density lipoprotein-cholesterol (HDL-C), apoprotein A (apoA), apoprotein B (apoB), lipoprotein (a) (Lp(a)) and glycated hemoglobin (HbA1c). Plasma level of Lp-PLA2 at admission was evaluated by enzyme-linking immune-absorbent assay (ELISA, commercially brought from Shanghai Yueyan Biological.

Technology Co.,Ltd , and the inter-assay variant is less than 11% and intra-assay variant is less than 9%) and the assay range was 1 μg/L to 20 μg/L. All performances were conducted in accordance to manufacture’s instruction and three independent experiments were performed in duplicate. For the purpose of figuring out whether increased plasma level of Lp-PLA2 was associated with the severity of CAD, all participants were divided into different subgroups on the basis of two major categories in terms of the severity of clinical presentation (stable angina, unstable angina, or acute myocardial infarction) and the number of stenosis coronary artery (single or multiple vessels stenosis).

### Studied endpoint

The primary endpoint of our current study was to evaluate the correlation between plasma level of Lp-PLA2 and the severity of CAD, including the severity of clinical presentation and the number of coronary artery stenosis.

### Statistical analyses

Continuous data was presented as mean ± SD or median (inter-quartile range) appropriately, and compared by the Student’s t-test when data was normally distributed, otherwise compared by the Wilcoxon rank-sum test. Categorical data was presented as percentage and compared by χ^2^ test. Univariate and multivariate regression analyses were performed to evaluate the relationship between Lp-PLA2 value and the severity of CAD. Statistical analyses were performed by using SPSS software version 18.0 (SPSS, Inc., Chicago, Illinois). A value of P < 0.05 was considered significant.

## Results

### Baseline characteristics of studied subjects

Totally 678 participants were angiographically diagnosed as CAD and 103 were ruled out of CAD and data was shown in Table 1. In comparison to the controls, participants with documented CAD were with more traditional risk factors. Briefly, participants with CAD were more elderly and with more prevalent of smoking and hypertension. Serum Lp(a) level was significantly higher while HDL-C and apoA level were profoundly lower in participants with CAD as compared to the controls, and importantly the changes of lipid profiles were associated with the severity of CAD. Notably, plasma level of Lp-PLA2 was gradually elevated in parallel with the increased severity of CAD. There was no significant difference among other variables between these groups. Briefly, the percentages of participants underwent statins treatment before enrollment in the control, SA, UA and AMI groups were 36.8%, 34.6%, 41.5% and 40.9% (P = 0.031) respectively, and in the control, single and multiple vessels stenosis groups were 36.8%, 40.2% and 41.7% (P = 0.036).

**Table 1:** **Baseline characteristics of participants**

**Group**	**Control**	**SA**	**UA**	**AMI**	**P**
**N**	103	190	293	195	
**Age (years)**	60.57 ± 9.57	62.77 ± 10.68	64.70 ± 10.83	61.79 ± 11.84	0.002
**Male (%)**	67(65.0)	154 (81.1)	225(76.8)	159(81.5)	0.006
**Family history (%)**	8(7.8)	12 (6.3)	12(4.1)	6(3.1)	0.216
**Smoking (%)**	25(24.3)	73 (38.4)	105(35.8)	87(44.6)	0.006
**Hypertension (%)**	67(65.0)	108 (56.8)	191(65.2)	103(52.8)	0.025
**Diabetes mellitus (%)**	24(23.3)	61 (32.1)	93(31.7)	60(30.8)	0.396
FBG (mmol/L)*	5.41 (3.78-13.64)	5.64 (5.06-6.94)	5.74(4.97-7.49)	5.85(4.92-8.22)	0.148
**HbA1c (%)**	6.33 ± 1.59	6.50 ± 1.37	6.44 ± 1.25	6.48 ± 1.38	0.727
Lp(a)(mmol/L)*	96.05 (27.06-158.56)	130.15 (66.79-293.25)	172.97(82.43-420.94)	176.97(101.09-289.26)	< 0.001
**TC(mmol/L)**	4.54 ± 1.07	4.29 ± 1.01	4.41 ± 1.22	4.43 ± 1.08	0.316
**TG(mmol/L)**	1.74 ± 1.40	1.59 ± 0.97	1.60 ± 0.95	1.64 ± 1.13	0.792
**LDL-C (mmol/L)**	2.72 ± 0.89	2.57 ± 0.86	2.67 ± 0.98	2.79 ± 0.91	0.263
**HDL-C (mmol/L)**	1.08 ± 0.26	1.02 ± 0.27	1.00 ± 0.25	0.90 ± 0.17	< 0.001
**apoA(mmol/L)**	1.18 ± 0.24	1.14 ± 0.32	1.10 ± 0.25	0.98 ± 0.20	< 0.001
**apoB(mmol/L)**	0.81 ± 0.18	0.77 ± 0.19	0.80 ± 0.22	0.85 ± 0.28	0.071
Lp-PLA2 (μg/L)*	7.37 (2.34-15.78)	5.94(2.89-8.55)	8.56(5.34-11.95)	8.68(5.56-13.27)	< 0.001

Denote: *indicated median and inter-quartile range, FBG = fasting blood glucose, HbA1c = glycated hemoglobin, Lp(a) = lipoprotein (a), TC = total cholesterol, TG = triglyceride, LDL-C = low density lipoprotein-cholesterol, HDL-C = high density lipoprotein-cholesterol, apoA = apoprotein A, apoB = apoprotein B, Lp-PLA2 = lipoprotein associated-phospholipase A2.

### Relationship between Lp-PLA2 level and severity of clinical presentation

Participants were divided into 4 groups in accordance to the severity of clinical presentation. Plasma level of Lp-PLA2 in the control (n = 103), SA (n = 190), UA (n = 293) and AMI (n = 195) groups were 7.38(3.33-9.26) μg/L, 5.94(2.89-8.55) μg/L, 8.56(5.34-11.95) μg/L and 8.68(5.56-13.27) μg/L respectively (AMI group versus control group, P < 0.001). After adjusted for age, gender, smoking, diabetes mellitus, hypertension, Lp(a), LDL-C, HDL-C, apoA and statins, Lp-PLA2 level was still independently associated with severity of CAD, with odd ratio (OR) of 1.055 (AMI versus control group, 95% confidence interval (CI) 1.021-1.090, P < 0.05).

### Relationship between Lp-PLA2 level and the number of coronary artery stenosis

In light of angiography findings, participants were divided into control, single, or multiple vessels stenosis groups. Lp-PLA2 levels in the control (n = 103), single-vessel stenosis (n = 193), and multiple-vessels stenosis (n = 485) groups were 7.38(3.33-9.26) μg/L, 7.80(4.05-10.76) μg/L, and 8.29(5.18-11.76) μg/L respectively (P for trend < 0.001). After adjusted for age, gender, smoking, diabetes mellitus, hypertension, Lp(a), LDL-C, HDL-C, apoA and statins, Lp-PLA2 level remained independently associated with the number of coronary artery stenosis, with OR of 1.053 (multiple-vessels stenosis group versus control group, 95% CI 1.025-1.069, P < 0.05).

## Discussion

Owing to its unique effects on the initiation and progression of atherosclerosis [18,19], Lp-PLA2 has been recognized as a novel and promising biomarker for cardiovascular diseases risk evaluation, and data from clinical and basic research also showed that Lp-PLA2 decrease was beneficial for retarding atherosclerosis progression and reducing cardiovascular events [10,20]. Nevertheless, results from two recent clinical trials named STABILITY and SOLID-TIMI52 did not show cardiovascular benefits of darapladib therapy in patients with stable or unstable CAD [16,17]. Consistent with previous epidemiological studies, findings from our current study shows that in the real world population increased plasma level of Lp-PLA2 is not only independently associated with more vessels stenosis but also portends a higher risk of cardiovascular events such as acute coronary syndrome.

Lp-PLA2 is an enzyme predominantly excreted from atherosclerotic plaques by inflammatory cells and circulates in blood stream. Pathologically, Lp-PLA2 augments inflammation and oxidation by means of degrading oxidized-LDL into two potent pro-atherosclerotic and pro-thrombotic intermediates named lysophosphotidylcholine (Lyso-PC) and oxidized non-esterified fatty acids (oxNEFAs), which subsequently results in endothelium dysfunction, foam cells formation, necrotic lipid-core expansion and fibrous cap thinning [21]. Therefore, it is reasonable to speculate that increased plasma level of Lp-PLA2 may contribute coronary artery stenosis progression and accelerate atherosclerotic plaque rupture. Data from current cross-sectional study also supported this hypothesis. As compared to those without significant coronary artery stenosis (control group), plasma level of Lp-PLA2 was gradually and significantly increased in single and multiple vessels stenosis groups. Previously, data from Shahar Lavi and colleagues also showed that early coronary atherosclerosis in humans was characterized by Lp-PLA2 production, and local coronary production of Lyso-PC, the active product of Lp-PLA2, was associated with endothelial dysfunction and atherogenesis [22]. Therefore, it might be possible that the higher Lp-PLA2 level the more severe of endothelial dysfunction and atherosclerosis would be. Although our current study has not tested the differences of endothelial function in each group, accumulating evidence from basic and clinical research might be compelling to demonstrate the positive relationship between increased Lp-PLA2 level and the impairment of endothelial function. Apart from endothelium-associated mechanisms, Lyso-PC and oxNEFAs also play important and complex roles on atherosclerotic plaques development [23]. Pathologically, both Lyso-PC and oxNEFAs are capable of up-regulating intra-cellular adhesion molecules (ICAM) expression, enhancing inflammatory cells infiltration and accumulation, promoting foam cells formation and expanding necrotic lipid-core [24]. Overall, Lp-PLA2 per se and its product concomitantly initiate and accelerate atherosclerotic plaque development.

Of note, serum level of Lp(a) was significantly higher in the AMI group than that of the control group, while HDL-C and apoA were profoundly lower, suggesting that these dyslipidemia might be also associated with the CAD severity. However, after adjustment for these lipid profiles, Lp-PLA2 level remained independently associated with CAD severity. With regard to the anti-inflammation, anti-oxidation and endothelium-protective effects, one might speculate that statins might be capable to ameliorate the adverse effects resulted from Lp-PLA2 elevation. Nonetheless, data from previous studies showed the conflicting outcomes of statins therapy on Lp-PLA2 elevation. Present research showed no confounding effects of statins therapy on the relationship between Lp-PLA2 level and CAD severity.

On the second critical point, results from our study supported the notion that increased plasma level of Lp-PLA2 predicted a more unstable phenotype of coronary artery plaques. Of note, plasma levels of Lp-PLA2 were comparable in the control and stable angina groups (7.38(3.33-9.26) μg/L and 5.94(2.89-8.55) μg/L, respectively). Nonetheless, Lp-PLA2 levels were significantly elevated in the unstable angina and acute myocardial infarction groups (8.56(5.34-11.95) μg/L and 8.68(5.56-13.27) μg/L, respectively). This finding was consistent with previous reports [14,25]. Data from Frank D. Kolodgie et al. showed that Lp-PLA2 staining was more intense in human prone-rupture coronary atheroma than that with relatively stable plaque [25]. Histologic and pathological results from basic research further supported clinical findings. The landmark research used hypercholesterolemic and diabetic swine showed that Lp-PLA2 played continuous role on coronary atherosclerotic plaque development [14]. Darapladib treatment resulted in a considerable decrease in necrotic core area and reduced medial destruction, thereby resulting in relatively stable phenotype of plaques, which indirectly suggested that Lp-PLA2 promoting plaque instability. Of note that plasma levels of Lp-PLA2 were significantly higher in the UA and AMI groups than that of the control and SA groups. To our best knowledge, it might be due to Lp-PLA continuously releasing from unstable prone-rupture or ruptured plaque. Therefore, we considered that increasing Lp-PLA2 level might portend an upcoming cardiovascular event such as acute coronary syndrome.

### Limitations

There were limitations of our current study. First of all, unavoidable bias of cross-sectional study exists with regard to evaluate the relationship between Lp-PLA2 level and severity of CAD. Nonetheless, traditional risk factors had already been extensively adjusted and previous abundant evidence also supported the positive relationship between increased Lp-PLA2 level and cardiovascular diseases risks. Secondly, the ELISA kit used in our current study maybe not consistent with previous use and the assay range was quite different from previous studies. Therefore it should be cautious to interpret our findings since the reference value of plasma level of Lp-PLA in our study was not consistent with previous studies. Nevertheless, three independent experiments were performed in duplicate, therefore we believed that the significant difference of Lp-PLA2 level among each subgroup was convincing. Last but not the least, previous studies had shown the relationship between Lp-PLA2 level and CAD severity and it appeared that our study provided not much new information. However, with respect to the conflicting results about Lp-PLA2 antagonist on cardiovascular outcomes, we considered that data from our study might add more evidence regarding the adverse effects of Lp-PLA2 on ASCVD, future studies selecting appropriate subjects was still deserved to conduct.

## Conclusion

Results from our study once again show that in patients with CAD, increased plasma level of Lp-PLA2 predicts an unstable phenotype of coronary atherosclerotic plaque and is also associated with more coronary artery stenosis. Dynamic evaluation of Lp-PLA2 level may provide prognostic value on cardiovascular diseases risk assessment.
